# Biogenic Amines: Signals Between Commensal Microbiota and Gut Physiology

**DOI:** 10.3389/fendo.2019.00504

**Published:** 2019-07-31

**Authors:** Nobuyuki Sudo

**Affiliations:** Department of Psychosomatic Medicine, Graduate School of Medical Sciences, Kyushu University, Fukuoka, Japan

**Keywords:** catecholamine, dopamine, gut microbiota, histamine, norepinephrine, serotonin

## Abstract

There is increasing interest in the interactions among the gut microbiota, gut, and brain, which is often referred to as the “microbiota-gut-brain” axis. Biogenic amines including dopamine, norepinephrine, serotonin, and histamines are all generated by commensal gut microorganisms and are suggested to play roles as signaling molecules mediating the function of the “microbiota-gut-brain” axis. In addition, such amines generated in the gut have attracted attention in terms of possible clues into the etiologies of depression, anxiety, and even psychosis. This review covers the latest research related to the potential role of microbe-derived amines such as catecholamine, serotonin, histamine, as well as other trace amines, in modulating not only gut physiology but also brain function of the host. Further attention in this field can offer not only insight into expanding the fundamental roles and impacts of the human microbiome, but also further offer new therapeutic strategies for psychological disorders based on regulating the balance of resident bacteria.

## Introduction

Humans co-exist with a vast number of microorganisms (1), and there has been increased research interest in uncovering the role of gut bacteria in maintaining health of the host. Accumulating evidence points to a major role of the gut microbiota in not only normal gut function but also in brain development and function (2–4). The recognition of such interactions between gut microorganisms and the brain has led to a new research field commonly referred to as the “microbiota-gut-brain” axis (5–9).

Biogenic amines are biogenic substances containing one or more amine groups (10, 11). Five of these amines were found to function as neurotransmitters including dopamine, norepinephrine, epinephrine, histamine, and serotonin. In general, these amines are utilized in the central and peripheral nervous systems, and participate in various types of physiological functions such as regulating cognitive abilities, mood, and gut motility (12–14). In addition to these well-established roles as neurotransmitters, accumulating evidence suggests that these amines might act as important signal molecules between commensal microbiota and the host in the gut (15–17). Here, the latest research progress on the relationship between gut microbiota and biogenic amines is reviewed, with a particular focus on interactions occurring in the gut lumen, in which gut bacteria are presumed to closely associate with biogenic amines.

## Catecholamines: Norepinephrine and Dopamine

### Catecholamines as Inter-Kingdom Signals in the Gut Lumen

Bacteria can communicate with each other through hormone-like signals in a process known as quorum sensing (18). Recent evidence has revealed that quorum sensing is not only restricted to bacterial cell–cell communication but also facilitates communication between microorganisms and their hosts. This type of bidirectional communication is often referred to as “microbial endocrinology” (15, 16) or “inter-kingdom signaling” (17, 19, 20), which thus mediates the symbiotic and pathogenic relationships between bacteria and their mammalian hosts.

Interestingly, catecholamines have emerged as potential inter-kingdom signaling molecules in the gut, in addition to their well-established roles as neurotransmitters in the central and peripheral nervous systems. In their pioneering studies conducted in the 1990s, Lyte et al. (21, 22) demonstrated that some pathogenic species could recognize exogenous catecholamines *in vitro*, which enhanced the bacterial proliferative capacity. Subsequently, Sperandio et al. (19) showed that enterohemorrhagic *Escherichia coli* virulence is increased upon exposure to epinephrine and norepinephrine, and that epinephrine binds and signals through the QseC receptor, providing a molecular basis for catecholamines acting as an inter-kingdom signal.

### Catecholamines Are Enriched in the Lumen of the Gut

Since a large number of bacteria inhabit areas in close proximity to the gastrointestinal tract of mammals, inter-kingdom signaling through catecholamines is presumed to continually and preferentially occur in the gut lumen. However, confirmation of this localized activity is a technical challenge because luminal samples generate substantial artifact peaks and contaminants, which hinder the precise evaluation of luminal catecholamine levels. In contrast, a clear and unique peak of 5-hydroxytryptamine (5-HT), or serotonin, was clearly identified without interference by contaminants (23). Therefore, to more reliably measure fecal catecholamine levels, we used a three-column high-performance liquid chromatography system with diphenylethylenediamine as a pre-column fluorescence derivatization reagent (24, 25), which was originally developed to evaluate plasma and urine free catecholamine levels. As a result, this analysis revealed considerable amounts of biologically active, free norepinephrine, and dopamine present in the gut lumen of specific pathogen free (SPF) mice, which harbor commensal gut microbiota that lacks specific pathogens (26).

The intestinal tract contains substantial amounts of β-glucuronidase (GUS) in both the gut epithelium (tissue type) and gut bacteria (bacterial type). The optimum pH of bacterial GUS is 6.8, whereas that of tissue-type GUS is 4.5 (27, 28). Since the average pH in the intestinal lumen is 6.5–8.0, GUS enzymatic activity is mainly derived from gut bacteria. GUS can play an important role in gut–liver interactions such as the so-called enterohepatic circulation (29–31). For example, hormones such as thyroid hormone are glucurono-conjugated in the liver, and the resultant conjugated materials are released into the bile duct. After reaching the lower gastrointestinal tract, these hormones are converted into biologically active hormones by the action of bacterial GUS, and thereafter reabsorbed into the body (30). Therefore, we examined whether biologically active free catecholamines can be produced by the action of bacterial GUS. As summarized (26), almost all catecholamines detected in SPF mice with a normal gut microbiota were of the biologically active free type (Table 1). In contrast, more than 90% of the dopamine and approximately 20 to 40% of the norepinephrine detected in germ-free (GF) mice were of the glucurono-conjugated type (Table 2). However, when GF mice were provided either a mixture of *Clostridia* species, a single bacterial species of *Clostridium coccoides*, or complete SPF microbiota rich in GUS activity, they exhibited a dramatic increase in free norepinephrine and dopamine (Table 3).

**Table 1:** Free and glucuronide-conjugated catecholamines in gut lumen of SPF mice^a^.

		**Free (ng/g)**	**Glucuronide–conjugated (ng/g)**
Ileum	norepinephrine	9.0 ± 2.1	3.8 ± 1.9
	dopamine	15.7 ± 4.1	44.9 ± 5.1^***^
Cecum	norepinephrine	34.6 ± 4.6	0.5 ± 0.2^***^
	dopamine	115.4 ± 14.4	1.6 ± 1.0^***^
Colon	norepinephrine	60.5 ± 6.0	2.0 ± 0.5^***^
	dopamine	177.0 ± 10.9	2.5 ± 0.7^***^

a *Luminal glucuronide-conjugated norepinephrine and dopamine levels in the gut lumen of specific pathogen-free (SPF) mice (n = 6) were measured using a three-column high-performance liquid chromatography system, as described in our report (26)*.

*** *P < 0.001 indicates significantly different from the corresponding free catecholamine value*.

**Table 2:** Free and glucuronide-conjugated catecholamines in gut lumen of GF mice^a^.

		**Free (ng/g)**	**Glucuronide- conjugated (ng/g)**
Ileum	Norepinephrine	7.5 ± 3.7	1.9 ± 0.3^*^
	Dopamine	1.5 ± 0.3	130.1 ± 16.3^***^
Cecum	Norepinephrine	3.8 ± 1.3	2.8 ± 0.6
	Dopamine	5.0 ± 0.5	136.9 ± 29.7^***^
Colon	Norepinephrine	3.2 ± 0.6	2.8 ± 0.6
	Dopamine	4.8 ± 0.3	138.0 ± 20.1^***^

a *Luminal glucuronide- and sulfate-conjugated norepinephrine and dopamine levels in the gut lumen of germ-free (GF) mice (n = 6) were measured as described in Table 1*.

* P < 0.05 and

*** *P < 0.001 indicates significantly different from the corresponding free catecholamine value*.

**Table 3:** Free catecholamine levels in the cecal lumen of gnotobiotic mice^a^.

	**GF mice**	**Gnotobiotic mice**		
		**Clostridia**	**Cc**	**EX-GF**
Norepinephrine (ng/g)	2.6 ± 0.3	27 ± 3^***^	82 ± 5^***^	18 ± 1^***^
Dopamine (ng/g)	16.3 ± 0.7	114 ± 9^***^	132 ± 16^***^	120 ± 16^***^

a *Cecal luminal contents collected from either germ-free (GF) (n = 6), Clostridia (n = 6), Clostridium coccoides (Cc, n = 6), or whole SPF microbiota (EX-GF, n = 6)-reconstituted mice were processed for free catecholamine measurements (26)*.

*** *P < 0.001 indicates significantly higher than the corresponding GF value*.

These results clearly indicate that gut microbes play a critical role in the production of free catecholamines through bacterial GUS.

### Can Commensal Bacteria Themselves Produce Catecholamines *in vivo*?

The next important question to address is whether gut microbes have the ability to generate catecholamines by themselves. Russian researchers (32) reported that some species of microorganisms could produce catecholamines in an *in vitro* culture system. Although this report is very interesting, the possibility that the medium used for bacterial culture contained catecholamines derived from blood, tissues, or other sources cannot be ruled out. To have the authentic capacity to synthesize catecholamines, microorganisms require tyrosine hydroxylase, a rate-limiting enzyme for catecholamine production. In fact, some bacteria were reported to possess enzymes that are similar to mammalian tyrosine hydroxylase (33, 34). In our study, the total norepinephrine levels in the cecal and colonic contents of SPF mice were substantially higher than those in GF mice (26). In addition, gut bacteria enriched from murine feces harbored substantial amounts of norepinephrine with a relatively lower amount of dopamine (35). These findings imply that gut microbes are an important source of luminal norepinephrine. However, some species of bacteria have a functional transporter for catecholamines such as the bacterial neurotransmitter sodium symporter family member, Leu T (36). Therefore, it remains to be clarified whether the norepinephrine and dopamine found in gut microbes originate from bacterial production via a tyrosine hydroxylase-like enzyme or if they are obtained from the gut lumen via a Leu T-like transporter (35). In this regard, Lyte and Brown (37) recently showed that *Lactobacillus salivarius* biofilms, but not *Lactobacillus rhamnosus* biofilms, might express both plasma membrane monoamine transporter- and organic cation transporter-like uptake systems, using specific fluorescence-based assays. These results indicate that bacteria residing in the gut lumen retain uptake systems that would act as a net sink for biogenic amines and neuroactive substances, and play an important role in preventing the excessive production of neurotoxic amines in the host.

### Functional Aspects of Catecholamines in the Gut Lumen

To reveal the specific roles that catecholamines play in the gut lumen, we further examined the effects of dopamine on water absorption in a mouse colon loop *in vitro* model (38, 39). As we previously reported (26), dopamine injection into the loop resulted in a 30% increase in water absorption from the gut lumen compared to that with vehicle (saline) injection, indicating that luminal dopamine can contribute to gut physiology under normal conditions. In addition, a recent report indicated the possible involvement of catecholamines in inflammatory bowel disease (IBD). Inhibiting catecholamine-QseC signaling attenuated disease activities in multiple preclinical IBD models (40), suggesting the therapeutic potential of QseC blockade for intestinal inflammation. Taken together, gut luminal catecholamines might contribute to a variety of as-yet-unidentified physiological and pathological functions.

## 5-HT

5-HT is abundant in the gastrointestinal tract, where it participates in various physiological functions such as peristalsis (41). The majority of 5-HT in the gut is stored in enterochromaffin cells (EC cells) and is secreted from the these cells into the gut lumen in response to various stimuli (42, 43). Intestinal bacteria are presumed to play a role in the secretion of 5-HT into the lumen; however, the precise underlying mechanism remains incompletely understood. Therefore, we examined kinetic 5-HT changes in the gut lumen after GF mice were provided SPF feces (23). As shown in Table 4, SPF fecal administration substantially elevated the cecal and colonic lumen 5-HT levels. In addition, approximately 50% of the 5-HT found in GF mice was in the conjugated form, whereas the majority of 5-HT found in EX-GF mice reconstituted with SPF feces was in the free form (Table 5). These results indicate that free 5-HT is not only released from EC stores in response to gut microbes but is also produced via the bacterial de-conjugation of conjugated 5-HT. Collectively, it can be concluded that free 5-HT in recolonized mice consists of the following three different components: (1) the first constitute is equivalent to free-5-HT in GF mice, which is likely released from EC cells in response to non-microbial stimuli; (2) the second is comparable to conjugated-5-HT in GF mice that is de-conjugated by microbial enzymes (GUS and sulfatase); (3) the third is mainly released from EC cells in response to microbial stimuli.

**Table 4:** Luminal 5-HT concentration in the gastrointestinal tract of GF mice after recolonization with SPF fecal microbiota^a^.

	**5-HT (ng/g)**			
	**Basal**	**Day 3**	**Day 7**	**Day 21**
Ileum	139 ± 106	218 ± 107	330 ± 183^*^	128 ± 116
Cecum	80 ± 20	535 ± 189^***^	547 ± 56^***^	381 ± 127^***^
Colon	230 ± 212	724 ± 198^***^	862 ± 230^***^	743 ± 241^***^

a *Luminal contents of germ-free (GF) mice were subjected to 5-HT measurements before (basal) and at 3, 7, or 21 days after exposure to specific pathogen-free (SPF) fecal microbiota (23)*.

* P <0.05 and

*** *P <0.001 compared to the corresponding basal value*.

**Table 5:** Luminal free and conjugated 5-HT in the colon of GF and EX-GF mice^a^.

	**Total 5-HT (ng/g)**	**Free 5-HT (ng/g)**	**Conjugated 5-HT (ng/g)**
GF	252 ± 89.0	106 ± 33.0	144 ± 75
EX-GF	563 ± 259^*^	501 ± 255^**^	62 ± 17^**^

a *Free and conjugated-5-HT levels were measured in the colonic lumens of germ-free (GF) and whole SPF microbiota-reconstituted (EX-GF) mice (n = 8) (23). Total 5-HT levels were calculated as the sum of free, glucuronide-conjugated, and sulfate-conjugated 5-HT*.

* P < 0.05 and

** *P < 0.01 compared to corresponding GF values*.

## Histamine and Other Biogenic Amines

Histamine is stored in mast cells and basophils and contributes to various types of patho-physiological processes. Histamine is best known as a mediator of the allergic reaction but also acts as a neurotransmitter for the brain (44). Histamine is not only synthesized in cells by the enzyme histidine decarboxylase but is also produced by the microbial decarboxylation of amino acids (45). This type of production is clinically important because bacteria-generated histamine often induces scombroid poisoning, which occurs after the consumption of food contaminated by amines (46). If an individual has a deficient capacity to detoxify biogenic amines due to genetic mutations or is taking antidepressants such as monoamine oxidase inhibitors (MAOIs) that slow the degradation of amines, they become more susceptible to histamine poisoning (46). Interestingly, commensal microorganisms in the gut can produce histamine and related compounds under physiological conditions (47, 48), suggesting the potential role of luminal histamine in gut immunoregulation. In fact, a recent elegant study demonstrated that histamine can exert an anti-inflammatory effects on the host by suppressing interleukin-18 production in the gut (49).

Moreover, researchers in this field have begun to turn their attention to the role of other biogenic amines such as tryptamine, phenethylamine, diamines (putrescine and cadaverine), and polyamines (spermine and spermidine) in mental health and psychiatric diseases (50, 51). In this regard, Gabastou et al. (52) offered an interesting case report. They measured time-course changes in fecal amines in a male patient who was admitted to a hospital due to severe brain damage incurred after a traffic accident at the age of 4 years. With a transient increase in these amines, the patient started to engage in self-injury and aggressive behaviors toward others. This case report indicates the possible involvement of bacteria-produced biogenic amines in the mental state. The authors suggested that antibiotics might be a suitable therapeutic option for psychomotor excitement that is refractory to commonly used treatments.

Thus, although the acute effects of histamine on the host, such as allergy and hypertension, are well established, the influence of histamine produced by indigenous microbes under physiological conditions remains unclear. This issue is especially relevant for not only individuals who are genetically susceptible to histamine exposure but also those who are regularly taking MAOIs for the treatment of depression.

## Biogenic Amine Receptors on Gut Epithelial Cells

Gut microbe-derived amines are presumed to exert a direct effect on gut epithelial cells via specific receptors. Here, we provide a literature review of the expression of receptors of biogenic amines, especially focusing on intestinal epithelial cells. As summarized in Table 6, the expression of α1-adrenergic receptor on enterocytes was demonstrated by flow cytometry and binding assays (53, 54). Enterocytes from different species were also shown to harbor α2 adrenergic receptors, contributing to the net absorption of electrolytes and fluids (55–57). Interestingly, rat EC cells possess both α1 and α2 receptors, which was verified by immunostaining and RT-PCR (58). Recently, intestinal stem cells were also reported to retain not only muscarinic acetylcholine but also α2 adrenergic receptors (59). In humans, RT-PCR analysis showed that β1 and β2 adrenergic receptors are present in the colon mucosa (60). Regarding dopamine receptors, their subtypes were confirmed in rat colonic epithelia (61) and goblet cells (62). Moreover, an excellent report using an advanced single-cell RNA sequencing technique recently demonstrated that Tuft cells express dopamine receptor D3 genes (63).

**Table 6:** Biogenic amine receptors detection in intestinal epithelial cells.

**Cell type**	**Receptors**	**Species**	**Assay methods**	**References**
Enterocyte	α1 Ad	Guinea pig	Flow cytometry, Fluorescent binding	(53)
Enterocyte (jejunum)	α1 Ad	Rat	Flow cytometry.	(54)
Epithelial cell (ileum)	α2 Ad	Human	Radioligand binding assay	(55)
Epithelial cell	α2 Ad	Rat	Radioligand binding assay	(56)
Enterocyte	α2 Ad	Human	Radioligand binding assay	(57)
EC cell	α1, α2 Ad	Rat	Immunostaining, RT-PCR	(58)
Intestinal stem cell	α2 Ad	Mouse	Quantitative real-time PCR	(59)
Colon mucosa	β1, β2 Ad	Human	RT-PCR	(60)
Colonic epithelial cell	D1A	Rat	Western blot, RT-PCR, *in situ* hybridization	(61)
Goblet cell	D1, D2, D3, D4, D5	Rat	IHC	(62)
Tuft cell	D3	Mouse	Single-cell RNA sequencing	(63)
Enterocyte, Paneth cell	5-HT2A	Mouse, rat, guinea pig	IHC	(64)
Colonic crypt cell	H1, H2	Dog	RT-PCR	(65)
Enterocyte	H1, H2, H4	Dog	IHC *in situ* hybridization	(66)
EC cell	TAAR1	Human	RT-PCR	(67)
Intestinal mucosal cell	TAAR1, TAAR2	Mouse	Quantitative real-time PCR	(68)

*EC, enterochromaffin; Ad, adrenergic; D, dopamine; H, histamine; TAAR, trace amine-associated receptor; RT-PCR, reverse transcription polymerase chain reaction; IHC, immunohistochemistry*.

Evidence as to whether the gut epithelia express receptors for other important monoamine molecules such as 5-HT and histamine is still scarce. One immunohistochemical analysis of different species demonstrated the presence of 5-HT2A receptors on enterocytes and Paneth cells (64). Histamine H1 and H2 receptors were also identified in canine enterocytes (65, 66); however, whether this expression is limited to dogs needs to be clarified. Finally, trace amine-associated receptors (TAARs) such as TAAR1 and TAAR2, for which ligands include tyramine, phenethylamine, and other biogenic amines (11), are also shown to exist in human EC cells (67) and the mouse intestinal mucosa (68). Since there is extensively increasing interest in trace amines derived from commensal bacteria, it is critically important to clarify the precise distribution and function of TAAR-related receptors in the gut. Finally, detailed information about the cellular localization of biogenic amine receptors, such as apical or basolateral membrane distributions, is still limited. This should be clarified by future studies.

## Conclusion and Perspectives

From the 19th to the early 20th century, a faction of scientists postulated that psychiatric diseases might result from “autointoxication,” suggesting that waste products or toxins generated in the gut can lead to depression, anxiety, and even psychosis (7, 69, 70). Until recently, the concept of autointoxication was regarded as an “unscientific” theory and was largely neglected. However, this theory has reemerged as an attractive research area and is currently being extensively studied. As reviewed herein, biogenic amines are interesting candidates that could be important mediators of autointoxication. Further developments in this field could provide a strong rationale for the application of probiotics for the treatment of mental health and diseases.

## Author Contributions

NS wrote the manuscript and contributed to all aspects of this paper.

### Conflict of Interest Statement

The author declares that the research was conducted in the absence of any commercial or financial relationships that could be construed as a potential conflict of interest.
